# Association between Sleep Duration and Urinary Albumin Excretion in Patients with Type 2 Diabetes: The Fukuoka Diabetes Registry

**DOI:** 10.1371/journal.pone.0078968

**Published:** 2013-11-12

**Authors:** Toshiaki Ohkuma, Hiroki Fujii, Masanori Iwase, Shinako Ogata-Kaizu, Hitoshi Ide, Yohei Kikuchi, Yasuhiro Idewaki, Tamaki Jodai, Yoichiro Hirakawa, Udai Nakamura, Takanari Kitazono

**Affiliations:** 1 Department of Medicine and Clinical Science, Graduate School of Medical Sciences, Kyushu University, Fukuoka, Japan; 2 Department of Environmental Medicine, Graduate School of Medical Sciences, Kyushu University, Fukuoka, Japan; 3 Department of Internal Medicine, Fukuoka Dental College Medical and Dental Hospital, Fukuoka, Japan; 4 Diabetes Center, Hakujyuji Hospital, Fukuoka, Japan; RIKEN Center for Integrative Medical Sciences, Japan

## Abstract

**Objective:**

Few studies have so far investigated the impact of sleep duration on chronic kidney disease in diabetic patients. The objective of the present study was to examine the relationship between sleep duration and albuminuria in type 2 diabetic patients.

**Research Design and Methods:**

A total of 4,870 Japanese type 2 diabetic patients ≥20 years of age were divided into six groups according to self-reported sleep duration: less than 4.5 hours, 4.5–5.4 hours, 5.5–6.4 hours, 6.5–7.4 hours, 7.5–8.4 hours and more than 8.5 hours. The association between sleep duration and urinary albumin-creatinine ratio (UACR) was examined cross-sectionally.

**Results:**

Both short and long sleep durations were significantly associated with higher UACR levels and higher proportions of patients with albuminuria (≥30 mg/g) and macroalbuminuria (≥300 mg/g) compared with a sleep duration of 6.5–7.4 hours (P for quadratic trend <0.001). A U-shaped association between sleep duration and UACR remained significant even after adjustment for potential confounders, including age, sex, duration of diabetes, current smoking habits, former smoking habits, current drinking habits, regular exercise habits, total energy intake, total protein intake, hypnotic use and estimated glomerular filtration rate. Furthermore, the association remained substantially unchanged after additional adjustment for body mass index, hemoglobin A_1c_, systolic blood pressure, renin-angiotensin system inhibitor use and depressive symptoms.

**Conclusions:**

Our findings suggest that sleep duration has a U-shaped association with the UACR levels in type 2 diabetic patients, independent of potential confounders.

## Introduction

In recent decades, the habitual sleep duration in most individuals has decreased [1], most likely owing to the transition from a traditional to a modern lifestyle. Epidemiological evidence suggests that a decreased sleep duration is associated with adverse consequences, such as obesity and weight gain [2], diabetes [3], [4], hypertension [5], cardiovascular diseases (CVD) [6] and increased mortality [7]. The negative impacts of a long sleep duration on these consequences have also been described [2]–[7], thus suggesting that there is a U-shaped relationship between sleep duration and adverse health outcomes.

Chronic kidney disease (CKD), a condition that is primarily characterized by proteinuria and/or a decreased glomerular filtration rate, has been established to be a predictor of end-stage renal failure and CVD and is therefore a major public health concern worldwide [8]. Proteinuria and albuminuria, even an increased urinary albumin-creatinine ratio (UACR) within the normal range, have also been shown to be associated with the development of both renal failure [9], [10] and CVD [11]–[13].

Recently, a possible association between a shorter sleep duration and proteinuria has been reported among Japanese people [14], [15]. However, to the best of our knowledge, there are no studies assessing the relationship between sleep duration and albuminuria, a more sensitive marker of CKD than proteinuria. Furthermore, no epidemiological studies have so far examined the association between sleep duration and either urinary albumin or protein excretion among diabetic patients, despite the fact that diabetes is the leading cause of end-stage renal failure and one of the most important risk factors for CVD. In this context, we investigated the association between sleep duration and urinary albumin excretion in Japanese type 2 diabetic patients.

## Methods

### Study Subjects

The Fukuoka Diabetes Registry is a multicenter prospective study designed to investigate the effects of modern treatments on the prognoses of diabetic patients regularly attending teaching hospitals certified by the Japan Diabetes Society and certified diabetes clinics in Fukuoka Prefecture, Japan (UMIN Clinical Trial Registry 000002627) [16]. A total of 5,131 diabetic patients 20 years of age or older were registered between April 2008 and October 2010. Diabetes mellitus was clinically diagnosed according to the criteria of the Japan Diabetes Society [17]. The exclusion criteria of the registry were: 1) patients with drug-induced diabetes or those undergoing steroid treatment, 2) patients under renal replacement therapy, 3) patients with serious diseases other than diabetes, such as advanced malignancies, decompensated liver cirrhosis, etc. and 4) patients unable to visit diabetologists regularly. After excluding 261 patients with type 1 diabetes, the remaining 4,870 patients (2,775 males, 2,095 females) were enrolled in this cross-sectional study. This study was conducted with the approval of the Kyushu University Institutional Review Board, and written informed consent was obtained from all of the participants.

### Clinical Evaluation and Laboratory Measurements

The participants completed a self-administered questionnaire covering their sleep duration, duration of diabetes, smoking habits, alcohol intake, physical activity level, diet and depressive symptoms. Sleep duration was self-reported and assessed based on answers to the question: “How long is your habitual sleep duration, including naps?” [18]. The participants were divided into six groups according to their sleep duration: less than 4.5 hours, 4.5–5.4 hours, 5.5–6.4 hours, 6.5–7.4 hours, 7.5–8.4 hours and more than 8.5 hours. Smoking habits were categorized current, former or never smoker. Alcohol intake was classified as either current or not. Participants engaging in sports regularly during their leisure time were defined as the regular exercise group. The dietary survey was conducted using a brief-type self-administered diet history questionnaire regarding the food frequency of 58 items (BDHQ; Gender Medical Research Inc., Tokyo, Japan). The validity of ranking the energy-adjusted intakes of many nutrients has been studied previously in an adult Japanese population [19], [20]. The presence of depressive symptoms was assessed using the Center for Epidemiologic Studies Depression Scale [21], and subjects who scored 16 or more out of 60 points were defined as having depressive symptoms. Body mass index (BMI) was calculated from each participant’s height and weight, and obesity was defined as BMI ≥30 kg/m^2^. Blood pressure was measured with the participant in the sitting position. Medication use was determined based on medical records. The participants were categorized as either taking oral hypoglycemic agents, insulin therapy, renin-angiotensin system (RAS) inhibitors, and hypnotic agents or not. Hypertension was defined as blood pressure ≥140/90 mmHg and/or current use of antihypertensive agents. Blood was collected via venipuncture. Hemoglobin A_1c_ (HbA_1c_) level was determined using high-performance liquid chromatography (Tosoh Corp., Tokyo, Japan). Serum creatinine was measured using an enzymatic method. The estimated glomerular filtration rate (eGFR) was estimated using the equation proposed by the Japanese Society of Nephrology [22] and given by the following equation: eGFR (ml/min/1.73 m^2^) = 194×[serum creatinine (mg/dl)]^−1.094^×[age (years)]^−0.287^×[0.739 if female]. A spot urine sample was obtained, and urinary creatinine and albumin were measured using an enzymatic method and an immunonephelometry method (Medical and Biological Laboratories Co., Ltd., Nagoya, Japan), respectively. The UACR (mg/g) levels were calculated by dividing the urinary albumin values by the urinary creatinine concentrations. Albuminuria was defined as UACR ≥30 mg/g, and macroalbuminuria was defined as UACR ≥300 mg/g.

### Statistical Analysis

Differences in the mean values or proportions of the characteristics of the studied subjects were tested by analysis of variance or chi-square test, as appropriate. The UACR values were presented as the median with 25th and 75th percentiles. The UACR values were log-transformed for the statistical analyses due to their skewed distribution, and compared using the Dunnett test. The rates of albuminuria and macroalbuminuria were compared using a logistic regression analysis. The age- and sex-adjusted or multivariate-adjusted partial regression coefficients and their 95% CIs of log-transformed UACR were determined using a multiple regression analysis. The quadratic trends of each value across the sleep duration categories were tested using a quadratic regression analysis. All analyses were performed using the SAS software package version 9.3 (SAS Institute Inc., Cary, NC). Values of P<0.05 were considered to be statistically significant for all analyses.

## Results

The clinical characteristics of the study participants are presented in Table 1 according to sleep duration. These characteristics were previously reported in part [18]. The mean age, the proportion of male patients and the duration of diabetes increased in association with an increase in sleep duration. The proportion of former smokers tended to increase, whereas that of never smokers tended to decrease as sleep duration became longer. The proportion of current drinkers exhibited no statistically significant differences among the sleep duration groups. Short sleepers were less likely to engage in regular exercise, had lower amounts of total energy and protein intake and were more likely to have depressive symptoms, hypnotic agents, higher BMI levels and to be obese. The HbA_1c_ levels increased in patients with a shorter or longer sleep duration as we previously reported [18]. The proportion of insulin users tended to increase with an increase in sleep duration. Although systolic blood pressure did not differ across the groups, diastolic blood pressure decreased and the proportion of patients taking RAS inhibitors increased in the long sleepers. The proportion of patients with hypertension increased in both the short and long sleepers. The eGFR was decreased in patients who slept longer.

**Table 1:** Clinical characteristics of the study subjects according to sleep duration.

	Sleep duration (h)						
	<4.5	4.5–5.4	5.5–6.4	6.5–7.4	7.5–8.4	≥8.5	P value
n	163	530	1228	1342	1141	466	
Age (years)	63±11	64±11	64±10	65±10	68±9	70±9	<0.001
Male (%)	44	49	55	57	61	67	<0.001
Duration of diabetes (years)	15±10	15±11	15±10	15±10	17±11	18±11	<0.001
Smoking habits							<0.001
Current smoker (%)	24	17	20	18	17	19	
Former smoker (%)	21	30	31	33	38	40	
Never smoker (%)	55	54	49	49	45	41	
Current drinker (%)	29	39	41	39	39	40	0.10
Regular exercise (%)	59	65	67	73	73	70	<0.001
Total energy intake (kcal/day)	1639±541	1631±518	1683±470	1705±502	1696±485	1716±517	0.03
Total protein intake (g/day)	65±25	65±26	67±23	68±24	68±24	69±26	0.12
Depressive symptoms (%)	26	13	7	7	8	9	<0.001
Hypnotic use (%)	25	12	12	10	11	10	<0.001
BMI (kg/m^2^)	25.3±4.4	24.8±4.5	23.9±3.8	23.6±3.8	23.4±3.4	23.4±3.5	<0.001
Obesity (%)	15	8	6	5	4	5	<0.001
HbA_1c_ (%)	7.71±1.18	7.52±1.11	7.44±1.02	7.39±1.04	7.40±1.01	7.46±1.07	0.002
OHA use (%)	70	65	65	63	64	61	0.30
Insulin use (%)	26	27	26	28	29	34	0.05
Systolic blood pressure (mmHg)	130±16	132±17	130±17	130±16	131±18	132±18	0.15
Diastolic blood pressure (mmHg)	74±10	75±10	75±10	75±11	74±11	73±11	0.02
RAS inhibitors use (%)	47	43	43	43	45	57	<0.001
Hypertension (%)	67	64	61	61	65	71	0.001
eGFR (ml/min/1.73 m^2^)	77±25	77±22	78±21	76±21	72±21	67±24	<0.001

The values are expressed as the mean ± SD or the percentage.

Abbreviations: BMI, body mass index; HbA_1c_, hemoglobin A_1c_; OHA, oral hypoglycemic agent; RAS, renin-angiotensin system; eGFR, estimated glomerular filtration rate.

Obesity was defined as BMI ≥30 kg/m^2^. Hypertension was defined as systolic blood pressure ≥140 mmHg or diastolic blood pressure ≥90 mmHg or the current use of antihypertensive agents.

**Figure 1:**
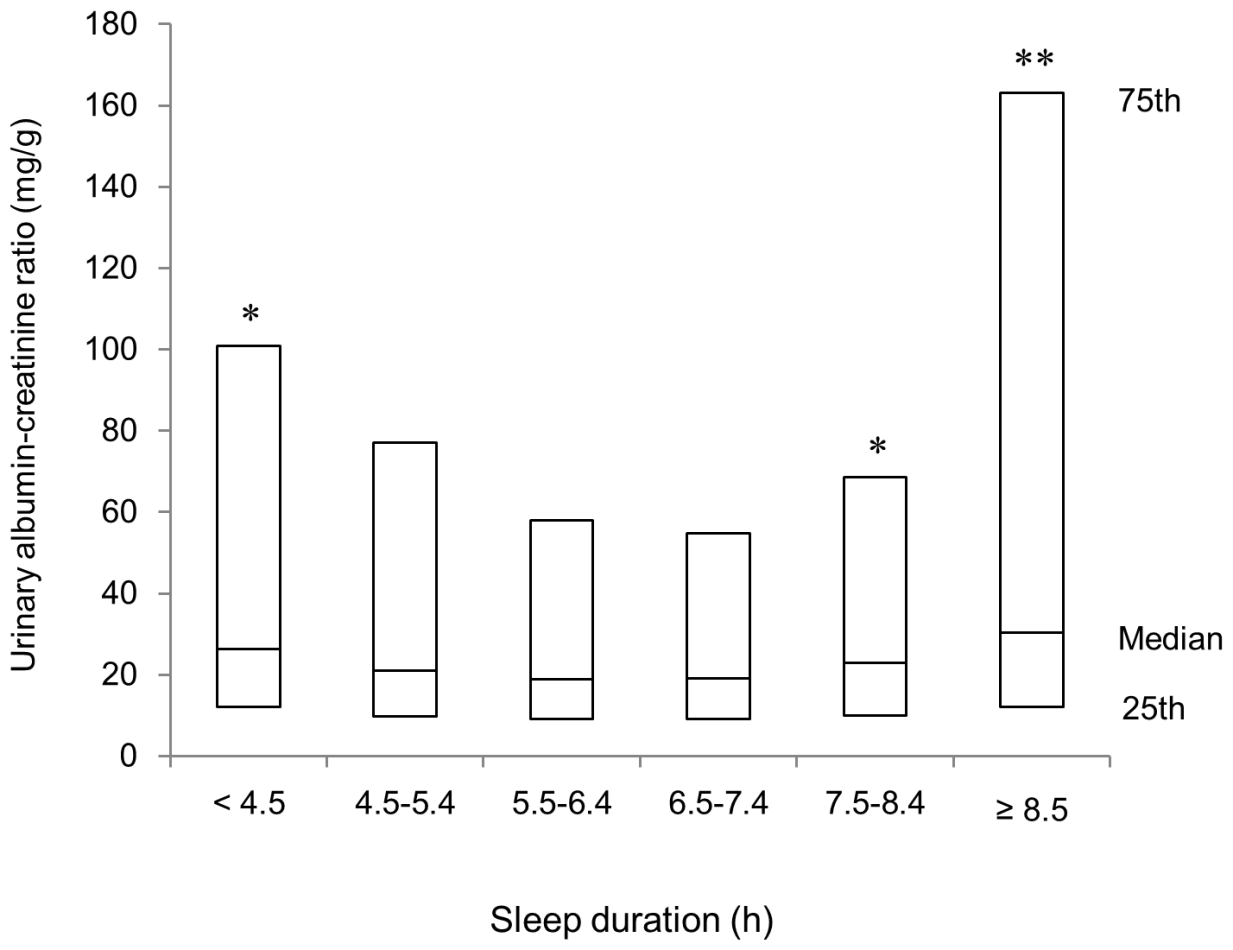
The percentile plot of the urinary albumin-creatinine ratio (UACR) levels according to sleep duration in Japanese type 2 diabetic patients. The bottom and top of the box are the 25th and 75th percentile, respectively, and the band in the box is the median. *p<0.05, **p<0.01 vs. sleep duration of 6.5–7.4 hours per day.

Figure 1 depicts the median values of UACR according to sleep duration, with a median level of 26.3 mg/g (25^th^–75th percentile, 12.1–101.0 mg/g) for patients sleeping less than 4.5 hours, 21.0 mg/g (9.7–77.1 mg/g) for patients sleeping 4.5–5.4 hours, 19.0 mg/g (9.1–58.1 mg/g) for patients sleeping 5.5–6.4 hours, 19.1 mg/g (9.0–54.9 mg/g) for patients sleeping 6.5–7.4 hours, 22.8 mg/g (10.0–68.7 mg/g) for patients sleeping 7.5–8.4 hours and 30.4 mg/g (12.0–163.2 mg/g) for patients sleeping more than 8.5 hours. Both patients with short and long sleep durations had higher log-transformed UACR levels compared with those who slept 6.5–7.4 hours (P for the quadratic trend <0.001), thus indicating the presence of an association occurring in a U-shaped fashion.

**Figure 2:**
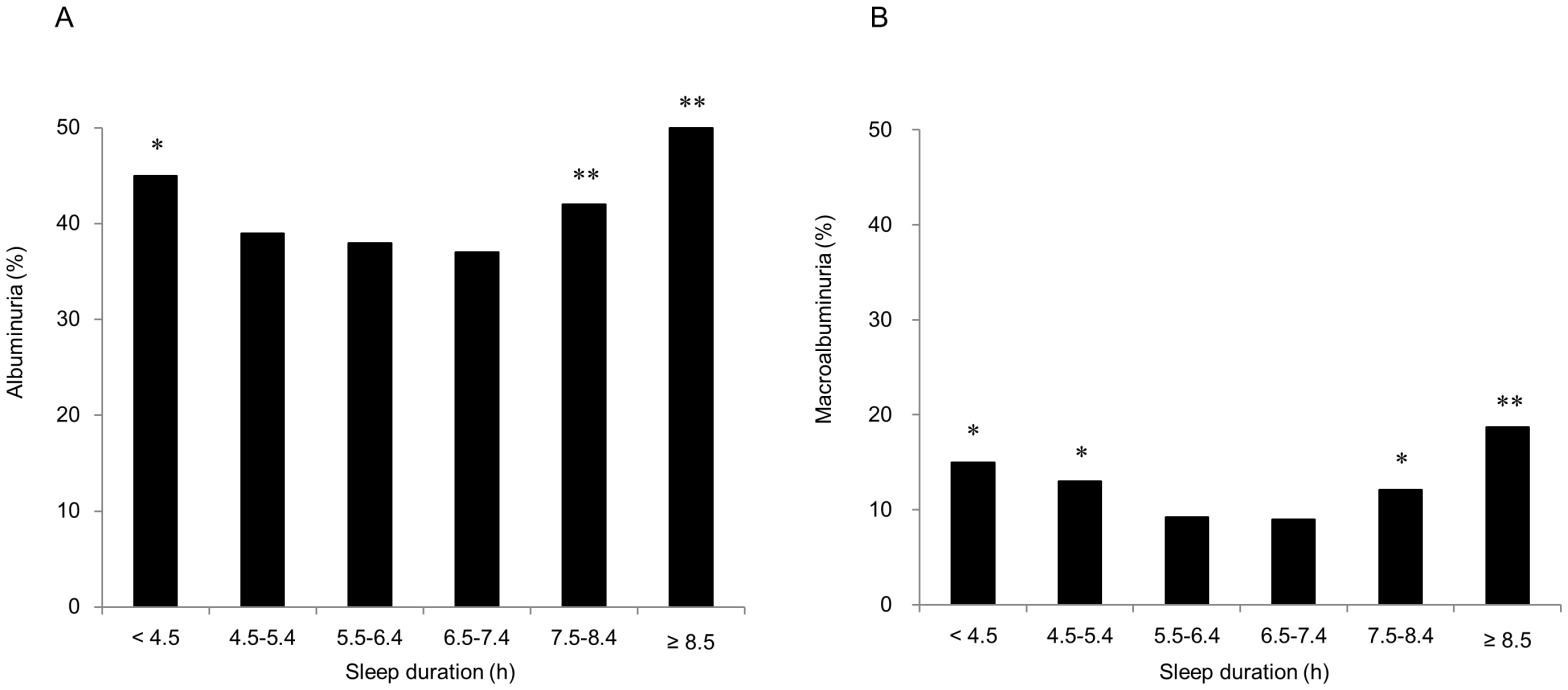
Proportions of patients with albuminuria (A) or macroalbuminuria (B) according to sleep duration in Japanese type 2 diabetic patients. Albuminuria was defined as urinary albumin-creatinine ratio (UACR) ≥30 mg/g, and macroalbuminuria was defined as UACR ≥300 mg/g. *p<0.05, **p<0.01 vs. sleep duration of 6.5–7.4 hours per day.

As shown in Figure 2, there were also U-shaped associations between sleep duration and the proportions of patients with albuminuria and macroalbuminuria (P for the quadratic trend <0.001 for both). The proportions of patients with albuminuria (Figure 2A) were 45%, 39%, 38%, 37%, 42% and 50% among the groups with a sleep duration of less than 4.5 hours, 4.5–5.4 hours, 5.5–6.4 hours, 6.5–7.4 hours, 7.5–8.4 hours and more than 8.5 hours, respectively. The proportions of patients with macroalbuminuria (Figure 2B) were 15%, 13%, 9%, 9%, 12% and 19%, respectively.

Table 2 shows the adjusted partial regression coefficients (95% CIs) of log-transformed UACR according to sleep duration. The UACR levels were increased significantly in patients sleeping for less than 4.5 hours, 4.5–5.4 hours or more than 8.5 hours in comparison with those observed in the patients sleeping for 6.5–7.4 hours (P for the quadratic trend <0.001) after controlling for age, sex, duration of diabetes, current smoking habits, former smoking habits, current drinking habits, regular exercise habits, total energy intake, total protein intake, hypnotic use and eGFR. These relationships were substantially unchanged following additional adjustment for BMI, HbA_1c_, systolic blood pressure, RAS inhibitor use and depressive symptoms. Furthermore, the sleep duration-UACR association persisted even after adjusting for these variables simultaneously, although the association was modestly attenuated. On the other hand, this relationship was not observed for eGFR following multivariate adjustment for the same factors (P for the quadratic trend = 0.11, the multivariate adjusted partial regression coefficients of 0.43 (95% CI −2.65, 3.51) for patients sleeping less than 4.5 hours, 0.58 (−1.31, 2.48) for 4.5–5.4 hours, 1.13 (−0.33, 2.58) for 5.5–6.4 hours, 0 (referent) for 6.5–7.4 hours, −0.13 (−1.62, 1.36) for 7.5–8.4 hours and −2.07 (−4.08, −0.06) for those sleeping more than 8.5 hours).

**Table 2:** Adjusted partial regression coefficients (95% CIs) of log-transformed UACR according to sleep duration.

	Sleep duration (h)						P for quadratic trend(across categories)	P for quadratic trend (continuous)
	< 4.5	4.5–5.4	5.5–6.4	6.5–7.4	7.5–8.4	≥ 8.5		
Age- and sex-adjusted	0.38	0.18	0.01	0	0.13	0.49	<0.001	<0.001
	(0.13, 0.64)	(0.02, 0.34)	(−0.11, 0.14)	(referent)	(0.01, 0.26)	(0.32, 0.66)		
Multivariate-adjusted	0.33	0.17	0.03	0	0.09	0.38	<0.001	<0.001
	(0.08, 0.57)	(0.02, 0.32)	(−0.09, 0.14)	(referent)	(−0.03, 0.21)	(0.22, 0.54)		
Multivariate-adjusted	0.25	0.11	0.01	0	0.08	0.36	<0.001	<0.001
+ BMI	(0.001, 0.49)	(−0.04, 0.26)	(−0.10, 0.13)	(referent)	(−0.03, 0.20)	(0.20, 0.52)		
Multivariate-adjusted	0.25	0.14	0.02	0	0.08	0.33	<0.001	<0.001
+ HbA_1c_	(0.01, 0.49)	(−0.01, 0.29)	(−0.10, 0.13)	(referent)	(−0.04, 0.20)	(0.18, 0.49)		
Multivariate-adjusted	0.29	0.14	0.02	0	0.10	0.32	<0.001	<0.001
+ SBP and RASI use	(0.06, 0.52)	(0.001, 0.29)	(−0.09, 0.13)	(referent)	(−0.02, 0.21)	(0.16, 0.47)		
Multivariate-adjusted	0.31	0.17	0.03	0	0.09	0.38	<0.001	<0.001
+ depressive symptoms	(0.06, 0.56)	(0.02, 0.32)	(−0.09, 0.14)	(referent)	(−0.03, 0.21)	(0.22, 0.54)		
Multivariate-adjusted+ BMI, HbA_1c_,	0.21	0.11	0.01	0	0.08	0.28	<0.001	<0.001
SBP, RASI use, and depressive symptoms	(−0.02, 0.44)	(−0.04, 0.25)	(−0.10, 0.12)	(referent)	(−0.03, 0.20)	(0.12, 0.43)		

The numbers in parentheses represent the 95% CIs.

Abbreviations: UACR, urinary albumin-creatinine ratio; BMI, body mass index; HbA_1c_, hemoglobin A_1c_; SBP, systolic blood pressure; RASI, renin-angiotensin system inhibitor.

Multivariate adjustment was made for age, sex, duration of diabetes, current smoking habits, former smoking habits, current drinking habits, regular exercise habits, total energy intake, total protein intake, hypnotic use and estimated glomerular filtration rate.

Finally, we performed sensitivity analyses after excluding 125 patients with an eGFR of less than 30 ml/min/1.73 m^2^ to eliminate the influence of renal dysfunction on urinary albumin excretion, and the results did not change significantly (data not shown).

## Discussion

The present study demonstrated that both short and long sleep durations are significantly associated with higher UACR levels and higher rates of albuminuria and macroalbuminuria compared with an intermediate sleep duration in Japanese type 2 diabetic patients. In this study, the U-shaped association between sleep duration and the UACR levels remained significant even after adjusting for confounding factors, namely, age, sex, duration of diabetes, current smoking habits, former smoking habits, current drinking habits, regular exercise habits, total energy intake, total protein intake, hypnotic use and eGFR. Moreover, the association remained substantially unchanged following additional adjustment for BMI, HbA_1c_, systolic blood pressure, RAS inhibitor use and depressive symptoms. To the best of our knowledge, this is the first large-scale epidemiological study to indicate a U-shaped curvilinear relationship between the sleep duration and the levels of urinary albumin excretion.

An increasing number of epidemiological studies have indicated the presence of U-shaped associations between sleep duration and various health disorders, such as an overweight status [2], diabetes [3], [4], hypertension [5], CVD [6] and mortality [7]. On the other hand, epidemiological evidence concerning a relationship between sleep duration and CKD is scarce. Recently, a self-reported short sleep duration (less than five hours) was found to be associated with proteinuria (OR 1.38 [95% CI 1.15–1.65]) in a cross-sectional study of Japanese employees [14]. In addition, a retrospective cohort study conducted among Japanese employees of a university hospital reported that subjects with a 5-hour or less self-reported sleep duration are at higher risk of developing proteinuria measured with dip-stick tests compared with subjects with a 7-hour sleep duration (incidence rate ratio 1.28 [95% CI 1.00–1.62] for subjects with 5 hours of sleep; 1.72 [95% CI 1.16–2.53] for those with ≤4 hours of sleep) [15]. However, it remains unclear whether this relationship exists in persons with a long sleep duration. Furthermore, the association between sleep duration and urinary albumin excretion, a more sensitive marker of CKD than proteinuria, has not been ascertained to date. The present study demonstrated that a shorter sleep duration is significantly associated with higher UACR levels, even after controlling for confounding factors. In addition, our findings also revealed an association between a longer sleep duration and higher UACR levels. Taken together, these findings indicate that there is a U-shaped relationship between sleep duration and albuminuria, thus implying that an inadequate sleep duration may have a negative impact on albuminuria, which has been shown to be a risk factor for both renal failure and CVD.

There are several potential mechanisms through which the relationship between an extreme sleep duration and albuminuria may be mediated. Short and long sleep durations have been reported to be associated with obesity [2], [18], hyperglycemia [3], [4], [18] and hypertension [5], as shown in the current study (Table 1). These disorders result in endothelial dysfunction [23]–[25] and glomerular hypertension [26], [27] and consequently lead to an increase in urinary albumin excretion. Depressive symptoms are also associated with both short and long sleep durations (Table 1), which may explain the association mediated via the increased inflammation [28] and sympathetic nervous system activation [29]. In a prospective study of hypertensive patients, a reduction in UACR achieved with antihypertensive therapy was smaller in patients with depressive symptoms than in those without, although a greater number of medications were required to achieve a similar blood pressure level at home [30]. This finding suggests the existence of an association between depressive symptoms and increased urinary albumin excretion. In the present study, however, the increased UACR levels observed in both short and long sleepers relative to moderate sleepers remained statistically significant, even after controlling for depressive symptoms and CVD risk factors, such as obesity, hyperglycemia and hypertension. These findings raise the possibility that sleep duration is associated with higher UACR levels independent of these risk factors.

Regarding a short sleep duration, systemic inflammation resulting from sleep loss may also account for the association with increased UACR levels. Sleep curtailment increases the levels of proinflammatory cytokines [31], high-sensitivity C-reactive protein [32] and white blood cells [33], all of which reflect systemic inflammation. Inflammation causes glomerular endothelial dysfunction and may consequently lead to albuminuria.

With respect to a long sleep duration, sleep-disordered breathing (SDB) may contribute to the association with increased UACR levels. Self-reported long sleepers (more than 9 hours) have a higher prevalence of snoring, a characteristic symptom of SDB, than normal-length sleepers (7–8 hours) [34]. Chronic intermittent hypoxia and sleep fragmentation may result in inflammation, oxidative stress and activation of the sympathetic nervous system and renin-angiotensin-aldosterone system, and, in turn, endothelial dysfunction and glomerular hypertension may increase urinary albumin excretion [35]. The prevalence of SDB has been shown to be high among diabetic patients (ranging from 58% to 86%) [36], and the severity of SDB is associated with increased urinary albumin excretion [37], [38]. Restless legs syndrome (RLS) is also associated with a self-reported long sleep duration compared with a normal sleep duration [34]. RLS is a condition characterized by unpleasant leg sensations that usually occur mostly during sleep and make individuals want to constantly move their legs. RLS is highly prevalent in type 2 diabetic patients (17.7%) compared with nondiabetic controls (5.5%) [39] and might lead to an increase in the UACR levels via activation of the sympathetic nervous system [40]. Another possibility is the confounding effects of other unmeasured factors or diseases that lead to an extended sleep duration. Further research on potential mechanisms mediating the effects of a long sleep duration on elevated UACR levels is needed.

The strengths of the current study include the enrollment of a relatively large number of type 2 diabetic patients, which allowed for the statistical power to detect differences and adjustment for potential confounders. Additional strengths include the uniform collection of urine samples and the use of a standardized method for measurement. Furthermore, in the current study, the UACR levels were determined using a quantitative method, while previous studies have assessed the presence of proteinuria using dip-stick tests. Therefore, the findings of the present study may have higher accuracy with regard to the relationship between sleep duration and urinary parameters than previous studies. Moreover, this is the first study to examine the UACR levels in relation to sleep duration in patients with type 2 diabetes.

Some limitations of our study should be noted. First, sleep duration was determined according to a self-reported questionnaire, as in many prior epidemiological studies, and was not measured objectively. However, several studies comparing self-reported sleep durations with those evaluated with objective methods have been reported [41], [42]. One study of 49 blind subjects reported a moderate correlation (r = 0.57) between self-reported and actigraphy-measured sleep durations [41]. Another study also indicated a moderate correlation (r = 0.45) by comparing self-reported sleep durations with those measured using actigraphy in over 600 healthy adults [42]. Second, we did not obtain information regarding the quality of sleep. Therefore, the presence of sleep disorders, such as SDB, may have potentially confounded the relationship, since we could not assess these disorders. Assuming a high prevalence of SDB among diabetic patients and a relationship with urinary albumin excretion [37], [38], the lack of measurements of SDB is a limitation. Third, UACR levels were determined by a single measurement, which may not be accurately representative of the status of the studied subjects. Fourth, protein content in last meal before sleep and the interval between last meal and the time going bed were not available, which may to some extent affect the UACR levels. Fifth, we obtained no information regarding nondiabetic causes of renal disease, such as chronic glomerulonephritis. The lack of this information may have reduced the accuracy of our findings to some extent. However, persons with these diseases may be likely to have albuminuria even if they do not have an extreme sleep duration. Given that this limitation can reduce the impact of an inadequate sleep duration on urinary albumin excretion, the true association may be stronger than that shown in the present study. Sixth, we cannot prove any cause-and-effect relationships due to the cross-sectional design of our study. Finally, there may be other confounding factors besides those evaluated in the present study.

In conclusion, the current study is the first epidemiological study to demonstrate the presence of a U-shaped association between sleep duration and urinary albumin excretion in patients with type 2 diabetes. Patients with either short or long sleep durations may be considered to have a high risk for both renal failure and CVD, given that increased urinary albumin excretion is a strong risk factor for these diseases. The present study indicates that the impact of sleep on the development of albuminuria merits further research.
